# Lufenuron can be transferred by gravid *Aedes aegypti* females to breeding sites and can affect their fertility, fecundity and blood intake capacity

**DOI:** 10.1186/s13071-020-04130-1

**Published:** 2020-05-15

**Authors:** Paula V. Gonzalez, Laura Harburguer

**Affiliations:** Centro de Investigaciones de Plagas e Insecticidas (CIPEIN-UNIDEF/CITEDEF/CONICET), J.B. de La Salle 4397, Villa Martelli (1603), Buenos Aires, Argentina

**Keywords:** *Aedes aegypti*, Lufenuron, Auto-disemination, Fertility, Fecundity

## Abstract

**Background:**

*Aedes aegypti* (L.) is the main vector of dengue, yellow fever, Zika and chikungunya viruses. A new method for controlling this mosquito has been developed based on the possibility that wild adult mosquitoes exposed to artificial resting sites contaminated with a larvicide, can disseminate it to larval breeding sites, is named “auto-dissemination”. The present study was undertaken to evaluate if a chitin synthesis inhibitor like lufenuron can be disseminated to larval breeding sites and prevent adult emergence and also if forced contact of *Ae. aegypti* females with treated surfaces can affect its fertility, fecundity, and blood intake capacity.

**Methods:**

Larval susceptibility to lufenuron was measured through EI_50_ and EI_90_. On the other hand, gravid females were exposed by tarsal contact to lufenuron-treated papers, we used the WHO susceptibility test kit tube to line the papers, and 1, 3 or 5 females for the transference. We also evaluated if the exposure of female mosquitoes to lufenuron-treated papers (0.4 and 1 mg a.i./cm^2^) has an effect on their fertility, fecundity or in the ability to feed on blood. In each assay 12–15 female mosquitoes were exposed to lufenuron for 1 h, 24 h before blood meal (BBM) or 24 h after a blood meal (ABM).

**Results:**

Lufenuron proved to be very active against *Ae. aegypti* larvae with an EI_50_ of 0.164 ppb and EI_90_ of 0.81 ppb. We also found that lufenuron can be transferred by females from treated surfaces to clean containers causing the inhibition of emergence of the larvae (between 30 and 50%). This effect was dependent on the concentration applied on the paper and the number of females added to each cage.

**Conclusions:**

This study introduces an innovation by first exploring the possibility that an insect growth regulator (IGR) belonging to the group of benzoylphenyl ureas, such as lufenuron, can be transferred by gravid females to breeding sites and that at the same time can have an effect on fertility, fecundity and blood intake capacity of adult mosquitoes.
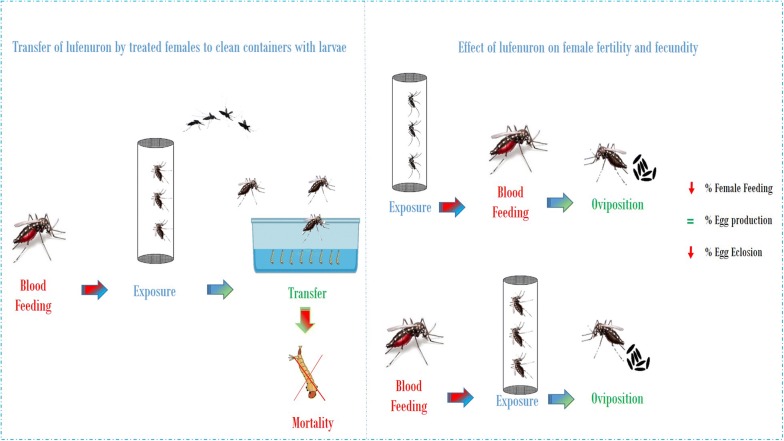

## Background

*Aedes aegypti* (L.) is the main vector of dengue, yellow fever, Zika and chikungunya viruses in many parts of the world affecting millions of people worldwide each year. The most commonly utilized strategy to reduce *Ae. aegypti* densities is aimed at the larval stages (removal of breeding sites, larvicide and community education) to reduce the population of new adults. Also, adult control using spatial sprays with adulticides is recommended when dengue outbreaks occurs [1]. Because adult emergence from container habitats is continuous, conventional adult insecticides spraying generally achieves inadequate and merely transient control [2–6].

The use of larvicides in containers that can result as potential breeding places and cannot be eliminated is the main alternative in control programmes, but this only targets an unknown percentage of the overall aquatic habitat. Application of larvicides to containers used as oviposition sites requires a house-to-house search to find and treat containers, which may not be feasible in large communities. In addition, treating larval production sites has produced insecticide-resistant populations especially to the principal larvicide used the last years, the organophosphorus temephos; pointing out the need of new larvicides for mosquito control [7–11].

During the past two decades, considerable progress has been made in the development of natural and synthetic compounds that are capable of interfering with the growth, development and reproduction processes of insects [12]. These substances are classified as insect growth regulators (IGRs) and compared with other insecticides, are safer for the environment and non-target organisms, including mammals, at the recommended doses [13–15]. There are three major groups of IGRs: the juvenile hormone analogues, the ecdysone agonists and the chitin synthesis inhibitors [12, 16]. A common property of this last group of IGRs, also called benzoylureas (BPU), is that they result in abortive molting and egg hatching as a consequence of chitin synthesis inhibition in the course of cuticle formation. The first chitin synthesis inhibitor introduced in markets was diflubenzuron [17]. This IGR was used successfully against various pest insects, including mosquitoes [14, 18, 19]. Among the most successful benzoylurea compounds next to diflubenzuron are triflumuron, hexaflumuron, lufenuron and novaluron. Lufenuron is one of the most newly introduced synthetic benzoylurea (CibaGeigy in 1998) used for the control of lepidopteran and coleopteran larvae. It is a compound found to be non-toxic to mammals and other vertebrates at the doses required against insects. Furthermore, it has been reported that lufenuron is suitable for integrated pest management programmes because of its long residual action and safety to adult beneficial insects, mites and spiders [20]. Although several reports describe the effects of different benzoylureas, against disease vectors [21–25], there is only one study that evaluates the toxicity of lufenuron on *Ae aegypti* larvae, nothing is known about its effect on the biology and reproductive fitness of the adult.

*Aedes aegypti* is a diurnal species that displays skip-oviposition behavior, i.e. lays small numbers of eggs in multiple sites [26]. These sites are often small and difficult to locate, which makes effective larvae elimination difficult. A novel method of control for this mosquito species was suggested from laboratory research results reported by Itoh et al. [27]. They found that blood-fed females of *Ae. aegypti* that had been forced into contact with surfaces treated with the IGR, pyriproxyfen (a juvenile hormone analogue), transported sufficient amounts to disrupt larval development in untreated oviposition sites. This approach, named “auto-dissemination”, is based on the possibility that wild adult mosquitoes exposed to artificial resting sites contaminated with pyriproxyfen, can disseminate insecticide to larval breeding sites, thus preventing adult emergence. Extraordinarily low doses of pyriproxyfen are needed to interfere with the metamorphosis of juvenile stages [28], and/or to cause morphological and functional aberrations in emerging adults, such as decreased fertility [29–31].

Since the work of Itoh et al. [27] substantial evidence has been collected in laboratory and field research showing that female mosquitoes either forced to walk on a pyriproxyfen-treated surface or topically contaminated can contaminate larval sites and significantly inhibit adult emergence [28, 32–35]. However, it has never been evaluated if an IGR belonging to chitin synthesis inhibitors group can be disseminated, which doses are needed on a contaminated surface to have an effect in untreated oviposition sites and if they can affect the adult fecundity and fertility.

The present study was undertaken to evaluate if a BPU like lufenuron can be disseminated to larval breeding sites and prevent adult emergence and also if forced contact of *Ae. aegypti* females with treated surfaces can affect its fertility, fecundity, and blood intake capacity.

## Methods

### Mosquitoes

A susceptible strain of *Ae. aegypti* (CIPEIN) was used. This strain originated from a Rockefeller strain from Venezuela and had been kept in the laboratory since 1996, reared at 26 ± 2 °C under a 12:12 h light:dark photoperiod. This colony is maintained free of exposure to pathogens, insecticides or repellents. Eggs were collected over wet cotton then dehydrated at room temperature and stored at least 30 days. They were rehydrated in dechlorinated water (about 500 eggs per 2 liters of water) and 24 h after rehydration, first-instar larvae were observed. Larvae were fed on a mixture of rabbit pellets and yeast. For this study, late third-instar or early fourth-instar larvae were used, and 5–7 day-old adults females.

### Chemicals

Lufenuron (N-[(2,5-dichloro-4-(1,1,2,3,3,3-hexafluoropropoxy)phenyl)carbamoyl]-2,6-difluorobenzamide), (technical grade 97.8%; Zhejiang Sega Science and Technology Co., Ltd, Huzhou City, China) was used. Silicone oil Dow Corning 556 was purchased from Daltosur SRL, Buenos Aires, Argentina. All solvents used (acetone) were analytical grade.

### Determination of larval susceptibility to lufenuron

The larvicidal bioassay was completed following the protocol by Bisset et al. [36]. One milliliter of the insecticide solution to be assayed was added to 224 ml of water in a 500 ml plastic container, and then was shaken lightly to ensure a homogeneous test solution. Then, 25 ml of water with 20 late third-instar or early fourth-instar *Ae. aegypti* larvae were added to the container. Five different concentrations of lufenuron were tested, ranging from 0.016 to 1 µg/l (ppb). One milliliter of solvent (acetone) was added to other cup and used as a control. Each concentration, including the control, was replicated five times. Cups containing the treated larvae were placed in a regulated chamber (26 ± 2 °C, 60–70% RH and 12:12 h photoperiod) with 100 mg of food mixture supplied once.

Bioassays were monitored for several days, until all insects either died or reached adulthood. Adult emergence inhibition (EI) data were registered as soon as all control group specimens emerged. The results were used to calculate the EI_50_ and EI_90_ values using the Probit method [37]. These values correspond to the lufenuron doses necessary to inhibit adult development of 50 and 90% of the specimens treated, respectively.

### Transfer of lufenuron by treated females to clean containers with larvae

Gravid females were exposed by tarsal contact to lufenuron-treated papers to determine if mosquitoes, after walking on the treated paper, could transport sufficient amounts to disrupt the development of larvae held in containers with water.

Methods modified from Itoh et al. [27] were used. Technical grade lufenuron was diluted with a 9:1 mixture of acetone:silicone oil and applied to Whatman® No. 1 filter paper (12 × 15 cm) at an application rate of 0.2, 0.4 and 1 mg active ingredient per cm^2^ (mg a.i./cm^2^). These doses were chosen based on a previous study using pyriproxyfen [32] where similar doses were used; also, knowing that lufenuron has a higher EI_50_/_90_ and therefore is less effective, a slightly higher dose was also added (1 mg/cm^2^). After acetone-silicone oil solutions of lufenuron were applied, paper discs were air-dried in the dark for 1 h and used to line the inside wall of a WHO susceptibility test kit tube and held in place by a wire ring.

Fifteen females, 5–7 days-old, were confined to the test kit for 1 h. They had been blood-fed 3 days before and on the day of the treatment. After exposure to treated paper 1, 3 or 5 treated females were released into a mosquito cardboard cage of 5 kg (13 cm height × 21.5 cm diameter) with gauze as lid. A plastic container (8 cm top diameter, 7 cm bottom diameter × 5 cm high) containing 20 late third-instar/early forth-instar larvae in 100 ml of water and lined with filter paper was added to the cage. The females were allowed to lay eggs for five days on the filter paper. The filter paper was then removed for observation of egg deposition. If any of the females in the cages retained eggs, the assay was discarded. Larvae in the plastic container were fed daily and reared at 26 ± 2 °C until adults emerged or all individuals die. Adult emergence inhibition (EI) data were calculated as described in the larvicidal bioassay.

Each combination of lufenuron dose and number of IGR-exposed females was replicated 5 times. Control papers were treated with 1 ml of the acetone-oil mixture, and used as described for IGR-treated papers.

Mosquito cardboard cages were discarded once each assay finished and new cages were used for the next replicates.

### Effect of lufenuron on female fertility and fecundity

As described before females were exposed by tarsal contact to lufenuron-treated papers (0.4 and 1 mg a.i./cm^2^) to determine if it has an effect on their fecundity (increase or decrease of the number of eggs laid), fertility (reduction of hatchability or viability of eggs) or in the ability to feed on blood.

In each assay 12–15 female mosquitoes were exposed to lufenuron for 1 h, 24 h before blood meal (BBM) or 24 h after a blood meal (ABM). Then females were released into a mosquito cardboard cage in which a container with 100 ml of water lined with Whatman® No. 1 filter paper (20 × 6 cm) as oviposition substrate was placed.

The females were allowed to lay eggs for 5 days and the number of eggs laid per female was counted, both on the filter paper and in the water. Females were removed and dissected, and the assay was discarded if retained eggs were found. The filter papers were left to dry and stored in sealed, plastic bags for 15 days to allow eggs to complete embryogenesis. After this period, a known number of eggs were placed in water to evaluate egg hatch and therefore, fecundity. As *Ae. aegypti* eggs usually hatch erratically or asynchronous depending on the environmental conditions [38], the number of larvae was counted after two, four and seven days.

For those females that were first exposed to lufenuron and then fed (BBM), we also evaluated the mortality 24 h after exposure and the percentage of females that fed on blood.

### Statistical analysis

In the determination of larval susceptibility to lufenuron bioassays, data were subjected to log-dose, probit-mortality analysis (PoloPlus 2.0 software LeOra Software Company, Petaluma, CA, USA). Prior to these analyses, the percentage inhibition of emergence of adults in treated containers was corrected for mortality in control containers [39].

Results of forced-contact experiments were analyzed by MANOVA (Sigmaplot 11.0, Systat Software Inc., San Jose, CA, USA) to determine if EI% varied significantly between trials, the number of females per bioassay cage or application rates. To compare the number of eggs/female, the percentage of hatched eggs and the females that feed on blood after exposure to lufenuron between the treated and control group a Student’s t-test was used, or a Mann–Whitney–Wilcoxon test when the assumption of normality was not met. The level of significance was set at *P* ≤ 0.05 (Sigmaplot 11.0).

## Results

### Transfer of lufenuron by treated females to clean containers with larvae

Lufenuron proved to be very active against *Ae. aegypti* larvae with an EI_50_ of 0.164 ppb (CI_95_ 0.039–0.486 ppb) and EI_90_ of 0.810 ppb (95% CI: 0.32–33.3 ppb) (Table 1).

**Table 1:** Toxicity of lufenuron to laboratory-reared late third-instar and early fourth-instar *Aedes aegypti* exposed continuously in the laboratory

Species	*n*	EI_50_ (ppb)	95% CI	EI_90_ (ppb)	95% CI	Slope	SE
*Ae. aegypti*	600	0.164	0.039–0.486	0.810	0.32–33.3	1.85	0.17

*Note*: EI_50_ and EI_90_ and 95% CI for lufenuron are expressed in µg/l (ppb)

*Abbreviations*: EI_50_, 50% adult emergence inhibition; EI_90_, 90% adult emergence inhibition; 95% CI, 95% confidence interval; SE, standard error

**Fig. 1:**
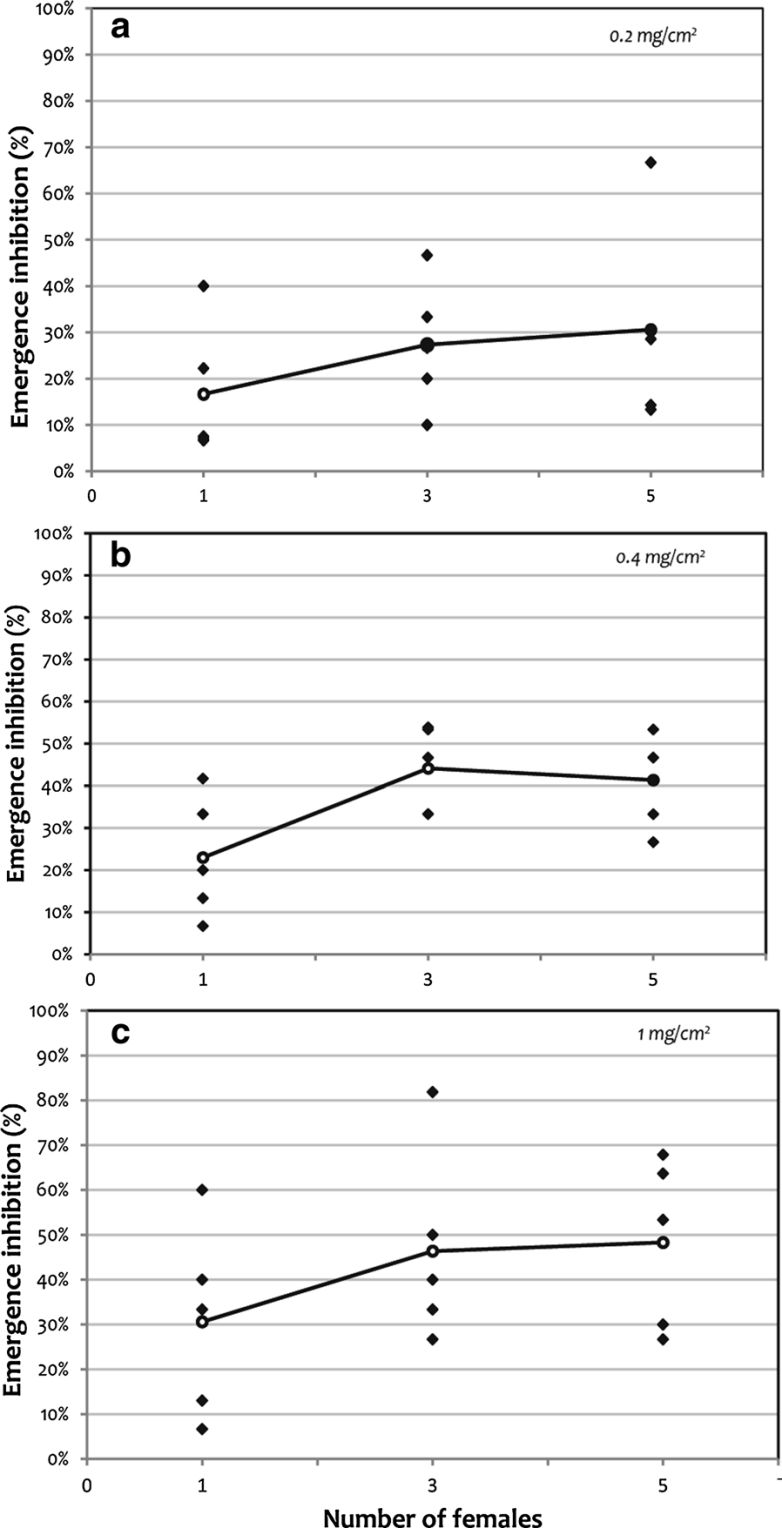
Lufenuron dissemination by gravid *Ae. aegypti* females to larval breeding sites measured as emergence inhibition (%). Emergence inhibition (%) achieved in larval microcosms through horizontal movement of lufenuron by gravid *Ae. aegypti* females that were forced to contact lufenuron-treated paper: 0.2 mg/cm^2^ (**a**), 0.4 mg/cm^2^ (**b**) or 1 mg/cm^2^ (**c**). Diamonds, data points; circles, means of five replicates

We also found that lufenuron can be transferred by females from treated surfaces to clean containers causing the inhibition of emergence of the larvae. This effect was dependent on the concentration applied on the paper (*F*_(2, 36)_ = 4.92, *P* = 0.013) and also the number of females (*F*_(2, 36)_ = 4.75, *P* = 0.015) added to each cage. When the lowest dose of lufenuron was applied (0.2 mg/cm^2^) there were no differences in the number of females used with a mean EI% between 17–30% (Fig. 1).

On the other hand, no differences in EI% were found between the doses applied to the paper when only one female was used to transfer lufenuron, indicating that there is a maximum amount that a female can transfer regardless of the dose with which she comes in contact (17, 23 and 30% EI for doses of 0.2, 0.4 and 1 mg/cm^2^, respectively). At the highest doses (0.4 and 1 mg/cm^2^) no differences were found between using 3 or 5 females. A slight tendency of higher larvae mortality values was observed in the dose of 1 mg/cm^2^ although on average they did not show significant differences with the dose of 0.4 mg/cm^2^.

### Effect of lufenuron on female fertility and fecundity

The effect of lufenuron on the immature stages of insects is well known; however little is known about its direct effect on adults. In this trial the effect of lufenuron on fertility, fecundity, and blood intake capacity of adult *Ae. aegypti* females was evaluated.

When females were exposed to lufenuron 24 h after blood meal (ABM) no differences were found in the number of eggs laid per female between the treated (0.4 or 1 mg/cm^2^) and the control groups (Table 2). No significant differences were found in the hatching percentage between the control group (82.7%) and the females that were exposed to a dose of 0.4 mg/cm^2^ (78.5%). However, significant differences were found in the group in which females contacted a surface with 1 mg/cm^2^ of lufenuron (83.1% in the control group *vs* 44.8% in the treated group; *t* = 3.42, *df* = 4, *P* = 0.026).

**Table 2:** Effects of IGRs lufenuron on egg production, hatchability of eggs, mortality and proportion of blood-fed for *Aedes aegypti* females

	No. of eggs/female ± SE		Hatching (%) ± SE		Mortality (%) ± SE		Females fed (%) ± SE	
	Control	Treated	Control	Treated	Control	Treated	Control	Treated
BBM								
0.4 mg/cm^2^	54.2 ± 4.4	48.2 ± 2.5	89.9 ± 4.4	79.5 ± 7.5	2.9 ± 1.8	5.1 ± 2.1	89.2 ± 6.7	63.6 ± 16.1
1 mg/cm^2^	46.9 ± 3.8	38.4 ± 10.9	88.3 ± 4.6	46.6 ± 20.0*	2.9 ± 1.8	7.0 ± 5.3	97.0 ± 2.0	56.2 ± 19.9*
ABMABM								
0.4 mg/cm^2^	49.6 ± 6.5	49.2 ± 3.3	82.7 ± 2.1	78.5 ± 4.5	–	–	–	–
1 mg/cm^2^	54.8 ± 7.6	47.7 ± 4.2	83.1 ± 1.1	44.8 ± 11.2*	–	–	–	–

* Significant differences between treated and control group (*P* < 0.05)

*Abbreviations*: ABM, exposition to lufenuron after blood meal; BBM, exposition to lufenuron before blood meal; SE: standard error

When the females were first exposed to lufenuron and fed after 24 h of this exposure (BBM), no differences were found in the number of eggs laid per female between the treated (0.4 or 1 mg/cm^2^) and the control groups (Table 2). Also, no significant differences were found in the hatching percentage between the control group (89.8%) and the females that were exposed to a dose of 0.4 mg/cm^2^ (79.5%). However, significant differences were found in the group in which females contacted a surface with 1 mg/cm^2^ of lufenuron (88.3% in the control group *vs* 46.6% in the treated group; *W* = 26, *P* = 0.029).

Additionally, for the BBM treatment, no differences were found in the percentage of mortality after 24 h between the females who had contacted a lufenuron surface before blood meal in both the 0.4 mg/cm^2^ (5.1%) or 1 mg/cm^2^ (7%) dose and the control females (3%). Regarding the ability of females to blood-feed who had previously contacted a lufenuron-treated surface, we found that 63.6% of the females who contacted a surface with 0.4 mg/cm^2^ blood-fed, while in the control group this percentage was 89.2%. Although the difference between groups was greater than 20%, it was not statistically significant.

On the other hand, 56.2% of the females who contacted a surface with 1 mg/cm^2^ of lufenuron blood-fed, while in the control group this percentage was 97%, with significant differences between the two groups (*W* = 37.5, *P* = 0.039).

## Discussion

Future challenges in integrated pest management require the development of selective and environmentally safe pesticides along with new strategies to apply them. The more specifically these insecticides act, and the less are their adverse side effects on beneficial insects and the environment, the more they are appropriate to control arthropod pests. Insecticides with novel modes of action, such as chitin synthesis inhibitors, disrupt cuticle formation [40]; suppression of chitin deposition in treated insects often causes high mortality during molting [41].

The survivorship of mosquitoes is very important for production of progeny, development, and transmission of pathogens among the hosts. Several studies reported the effects of sublethal exposure of IGRs on survival, fecundity, fertility, and blood intake capacity of female mosquitoes [28–31, 42–45]. However, very few studies have evaluated the direct effect of IGRs, mainly pyriproxyfen, on adult mosquitoes [46–49].

Among the new strategies that are trying to be implemented to control vector mosquitoes is the approach called “auto-dissemination”, based on the possibility that wild adult mosquitoes exposed to artificial resting sites contaminated with IGRs (so far pyriproxyfen, a juvenile hormone analogue), can disseminate insecticide to larval breeding sites, thus preventing adult emergence [28, 30, 32, 33]. This strategy is facilitated by the oviposition behaviour of *Ae. aegypti*, that typically distribute the eggs from a single gonothrophic cycle among several temporary sites. The “auto-dissemination” approach can be proposed as a ‘pull’ (i.e. attraction of wild mosquitoes to contaminated sites) and ‘push’ (i.e. dispersal of contaminated mosquitoes and dissemination of IGR to larval habitats) control strategy with the potential to target the myriad of cryptic larval breeding sites that cannot be reached by traditional larvicidal applications.

The objective of our study was to evaluate for the first time if an IGR belonging to the chitin synthesis inhibitors group can be transferred by female mosquitoes who contact a treated surface to larval breeding sites. On the other hand, it was also studied whether contact with a lufenuron-treated surface can have effects on female’s fertility, fecundity, and their blood intake capacity.

In our study, lufenuron proved to be highly effective on *Ae. aegypti* larvae with an EI_50_ of 0.164 ppb and an EI_90_ of 0.810 ppb. These values were significantly lower than those found by Salokhe et al. [50], who obtained a value of 6 ppb; although these authors did not specify if they used technical grade lufenuron or a formulation thereby preventing a direct comparison with our results. The values obtained in our study are similar to those obtained for other IGRs of this group such as triflumuron [24] with a value of EI_50_ and EI_90_ of 0.8 and 1.8 ppb, respectively. Also, for diflubenzuron a value of 0.5 ppb and 3.5 ppb was obtained for the EI_50_ and EI_90_, respectively [44]. Although the EI_50_ values for pyriproxyfen, the IGR used in the “auto-dissemination” assays, are approximately 10 times smaller (EI_50_ = 0.011 ppb [27]), we believe that the values obtained for lufenuron indicate that it would be a good candidate to explore in future field trials.

Regarding the “auto-dissemination” or horizontal transfer of lufenuron, our study shows that female mosquitoes contaminated with lufenuron can transfer enough material to water containers to exert a significant lethal effect on larvae developing there. This effect was dependent on the concentration applied on the paper and the number of females added to each cage. To the best of our knowledge, this is the first study to demonstrate that an IGR of the BPU group can be transferred to larval breeding sites by female mosquitoes. On the other hand, there is ample evidence of this phenomenon for pyriproxyfen [20, 30, 31] were mortality is almost exclusively limited to the pupal stage [27]. However, the use of IGR of the BPU group may represent an advantage since mortality would occur earlier in development, that is, between larval molts or molting from larva to pupa.

The doses of lufenuron used in our study were slightly higher than those used in other studies with pyriproxyfen. This is due to the difference in effectiveness between both active ingredients (measured as EI_50_), and because the surfaces used in the studies using pyriproxyfen were different from the paper used in our study. For example, a film of polyethylene terephthalat was used in the study of Itoh et al. [27], while Dell Chism & Apperson [32] used seed germination papers. These surfaces could allow a greater bioavailability of the IGR, therefore, it would be interesting to repeat our experiments using different surfaces and evaluate if this could have any differential effect.

Itoh et al. [27] reported equivalent effects using 1, 3 or 5 *Ae. aegypti* females; however, Dell Chism & Apperson [32] using *Ae. albopictus*, found that the percentage mortality was significantly lower at a density of one female per cage and increased to higher and equivalent levels of mortality at 3 and 5 females. These authors attributed the difference to a variation in sensitivity between the two species. In our study, the results were similar to those of Dell Chism & Apperson’s study [32], probably because lufenuron is less potent as a larvicide than pyriproxyfen, the amount of females that are involved in the transfer of the IGR is important.

In our study the effect of lufenuron on fertility, fecundity, and blood intake capacity of adult *Ae. aegypti* females directly exposed to a surface treated with lufenuron was evaluated. When females were exposed to lufenuron 24 hours after a blood meal (ABM) a reduction in the hatching percentage close to 40% was observed in females that had contacted a surface treated with a dose of 1 mg/cm^2^. Also, when contact with the surface treated with the IGR was before blood-feeding (BBM), a 40% reduction in egg hatching was observed at the dose of 1 mg/cm^2^. This would indicate that the moment at which contact with lufenuron occurs (before or after blood-feeding) is not decisive for the effect on fertility and fecundity but the dose used is, since the same results were obtained with both ABM and BBM regimes. On the other hand, the studies developed with pyriproxyfen indicate that the moment of contact with this IGR is decisive for its effect on the fertility and fecundity of the adult mosquitoes. Aiku et al. [51] found a significant effect on egg hatching when *An. stephensi* females were exposed to a bednet treated with 2% pyriproxyfen 24 hours after blood-feeding. Also, Gaugler et al. [34] found the same results with *Ae. albopictus*, suggesting that mosquitoes are most susceptible when blood-fed one day prior to pyriproxyfen exposure and therefore exposed while developing their eggs. Itoh et al. [27] reported that tarsal contact of *Ae. aegypti* with 0.1 mg/cm^2^ pyriproxyfen before a blood meal induced a large decrease in the number of hatched eggs compared with contact after a blood meal.

Finally, our study showed a reduction in the percentage of females that fed on blood at a dose of 1 mg/cm^2^ of lufenuron, from 97% in the control group to less than 60% in the treated group. However, there was no effect of previous exposure to lufenuron on female mortality. There are no other studies that evaluate the direct effect of IGRs on the blood intake capacity of female mosquitoes. Only research by Vasuki [45] found that *Ae. aegypti* larvae and pupae exposed to sublethal doses of the IGR hexaflumuron significantly reduced the quantity of blood ingested as adult females with a corresponding reduction in egg laying.

## Conclusions

During the past decades, a large number of chemically unrelated insecticidal compounds have been developed and commercialized that interfere with chitin synthesis, which is essential to reproduction, growth and development of insects [52]. This study introduces an innovation by first exploring the possibility that an IGR belonging to the group of BPUs, such as lufenuron, can be transferred by gravid females to breeding sites, and that at the same time can have an effect on fertility, fecundity and blood intake capacity of adult mosquitoes. Although lufenuron is effective against many insects, the proposed approach targets container-breeding species with such tiny amounts of compound disseminated exclusively to their breeding sites, that the impact on non-target species is likely to be minimal. In the future, it would be interesting to explore lufenuron, in field conditions.

## Data Availability

Data supporting the conclusions of this article are included within the article. The datasets used and analyzed during the present study are available from the corresponding author upon reasonable request.

## Abbreviations

BPU: benzoylureas
EI: adult emergence inhibition
EI%: adult emergence inhibition percentage
EI_50_: adult emergence inhibition 50%
EI_90_: adult emergence inhibition 90%
95% CI: 95% confidence limits
ABM: after blood meal
BBM: before blood meal
